# Potential geographic displacement of Chagas disease vectors under climate change

**DOI:** 10.1111/mve.12810

**Published:** 2025-05-21

**Authors:** Leandro Schlemmer Brasil, Divino Vicente Silvério, José Orlando de Almeida Silva, Walter Souza Santos, Leonardo Viana de Melo, Leandro Juen, Filipe Machado França, Thiago Bernardi Vieira

**Affiliations:** ^1^ Instituto de Ciências Biológicas e da Saúde Universidade Federal de Mato Grosso Pontal do Araguaia Mato Gross Brazil; ^2^ Programa de Pós‐Graduação em Zoologia Universidade Federal do Pará, Instituto de Ciências Biológicas Belém Pará Brazil; ^3^ Departmento de Biologia Universidade Federal Rural da Amazônia (UFRA) Capitão Poço Pará Brazil; ^4^ Centro de Ciências de Codó, Curso de Licenciatura Interdisciplinar em Ciências Naturais/Biologia Universidade Federal do Maranhão Codó Maranhão Brazil; ^5^ Programa de Pós‐Graduação em Ciências Ambientais Universidade Federal do Maranhão Chapadinha Maranhão Brazil; ^6^ Laboratório de Epidemiologia das Leishmanioses Instituto Evandro Chagas Ananindeua Pará Brazil; ^7^ Laboratório de análises clínicas Hospital do câncer de Rio Verde Rio Verde Goiás Brazil; ^8^ Instituto de Ciências Biológicas, Programa de Pós‐Graduação em Ecologia Universidade Federal do Pará Belém Pará Brazil; ^9^ School of Biological Sciences University of Bristol Bristol UK

**Keywords:** climate change impacts, medical entomology, Neotropical region, spatiotemporal distribution of vectors, Triatominae

## Abstract

Climate change is projected to profoundly alter global biodiversity with significant implications for vector‐borne disease dynamics. In tropical regions, rising temperatures and shifting precipitation patterns influence the distribution and behaviour of insect disease vectors, thereby affecting disease transmission cycles. Chagas disease, caused by the *Trypanosoma cruzi* and transmitted by triatomine bugs, is a major public health concern in Latin America. Brazil is particularly vulnerable to climate‐driven vector redistribution due to its vast land area, diverse ecosystems and rapid land‐use changes. Using ecological niche modelling and 11,640 unique occurrence records, we assessed the potential geographic displacement of 55 triatomine species under two climate scenarios: a moderate warming scenario (SSP2‐4.5) and a high‐emissions scenario (SSP5‐8.5) for 2050 and 2080. While projections for 2050 suggest stability in vector distributions, our models indicate a substantial shift by 2080, with increasing suitability for vector populations in the Brazilian Amazon, particularly in the deforestation arc. This expansion could exacerbate Chagas disease risk in previously unaffected regions, where socioeconomically vulnerable populations face poor housing conditions that facilitate vector‐human contact. Our findings underscore the urgent need for proactive vector surveillance, public health interventions and climate‐adaptive disease prevention strategies to mitigate potential epidemiological risks associated with climate change.

## Graphical abstract

- As a result of modelling the potential distribution of 55 triatomine species in the Neotropical region, with climate change projected for up to 2050, we found no significant changes.
- With climate change projected for up to 2080 in the Neotropical region, we see a significant potential for the migration of 55 species of triatomines towards the Amazon.
- Preventive actions by governments and the population are necessary to avoid future contamination by pathogens that cause Chagas disease transmitted by infected triatomines in the Amazon.

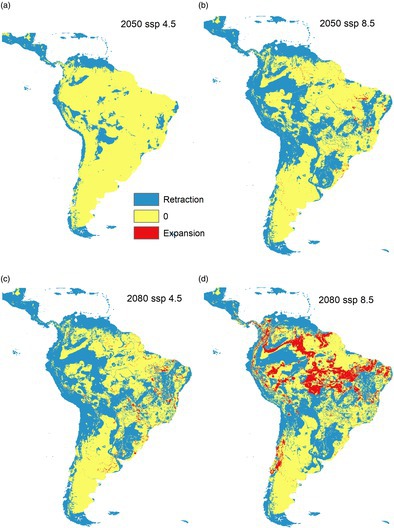

## INTRODUCTION

Climate change represents a major challenge to global food security (Gregory et al., 2005), water security (Stringer et al., 2021), the economy (Koubi, 2017) and public health (Cavicchioli et al., 2019). It is estimated that between 14% and 32% of species worldwide could face extinction due to climate change (Wiens & Zelinka, 2024). However, these impacts are not uniformly distributed in socioeconomically vulnerable regions, particularly tropical countries, which are expected to experience disproportionate consequences. Among these consequences, vector‐borne diseases are of particular concern because their transmission cycles are closely related to climatic variables and host species, such as mammals and insects (Byers et al., 2018; Ellwanger et al., 2020; Waha et al., 2017).

Rising temperatures and altered precipitation patterns are predicted to affect the distribution, abundance and behaviour of both vector and host organisms, leading to direct and indirect changes in the incidence of infectious diseases (Patz et al., 2003; Van de Vuurst & Escobar, 2023). Specifically, in tropical environments, warmer temperatures may accelerate the life cycles of insect vectors, enhance their reproductive rates and increase pathogen transmission efficiency (Yadav & Upadhyay, 2023). Brazil is particularly vulnerable to the impacts of climate change due to its extensive land area, diverse ecosystems and ongoing changes in land use and occupation (Byers et al., 2018; Coe et al., 2016). Current climate projections indicate temperature increases and precipitation reductions across multiple regions, modifying current climatic patterns and potentially affecting species distribution (Hobi et al., 2021; Phillips, 2008). For species with limited physiological tolerance, these changes may exceed their adaptive capacity, prompting shifts towards more climatically suitable regions (Broennimann et al., 2006).

However, species redistribution does not occur under an ecological vacuum. When species relocate, they interact with pre‐existing communities and organisms, potentially affecting their ecological relationships (Lawlor et al., 2024). A recent study in North America documented historical and contemporary shifts in the spatial distribution of vector insects (Santos et al., 2023), highlighting the importance of monitoring such changes for disease control. In Brazil, where over 203 million people reside, approximately 25% live in inadequate housing conditions (IBGE, 2023)—a well‐established risk factor for public health and vector‐borne diseases, including Chagas disease (Chastonay & Chastonay, 2022; Keall et al., 2010). Poor housing conditions facilitate contact with triatomine bugs (Hemiptera: Reduviidae), commonly known as ‘kissing bugs’, which transmit the protozoan *Trypanosoma cruzi* (Kinetoplastida: Trypanosomatidae), the etiological agent of Chagas disease (Jurberg et al., 2014). Given that climate change may alter triatomine distribution, understanding these potential shifts is essential for future disease prevention strategies.

To address this knowledge gap, we compiled 11,647 unique triatomine occurrence records across Brazil and analysed how climate change may impact their potential distribution at an unprecedented spatial scale. We applied ecological niche modelling under two future climate scenarios: a moderate warming scenario (SSP2‐4.5) and a high‐emissions scenario (SSP5‐8.5). By comparing the present and future distributions, we aimed to assess whether triatomine vectors are likely to expand into new areas, contract from current ranges or remain stable, thereby providing insights into the future risk of Chagas disease in Brazil.

## MATERIALS AND METHODS

### Occurrence data and species modelling

**FIGURE 1:**
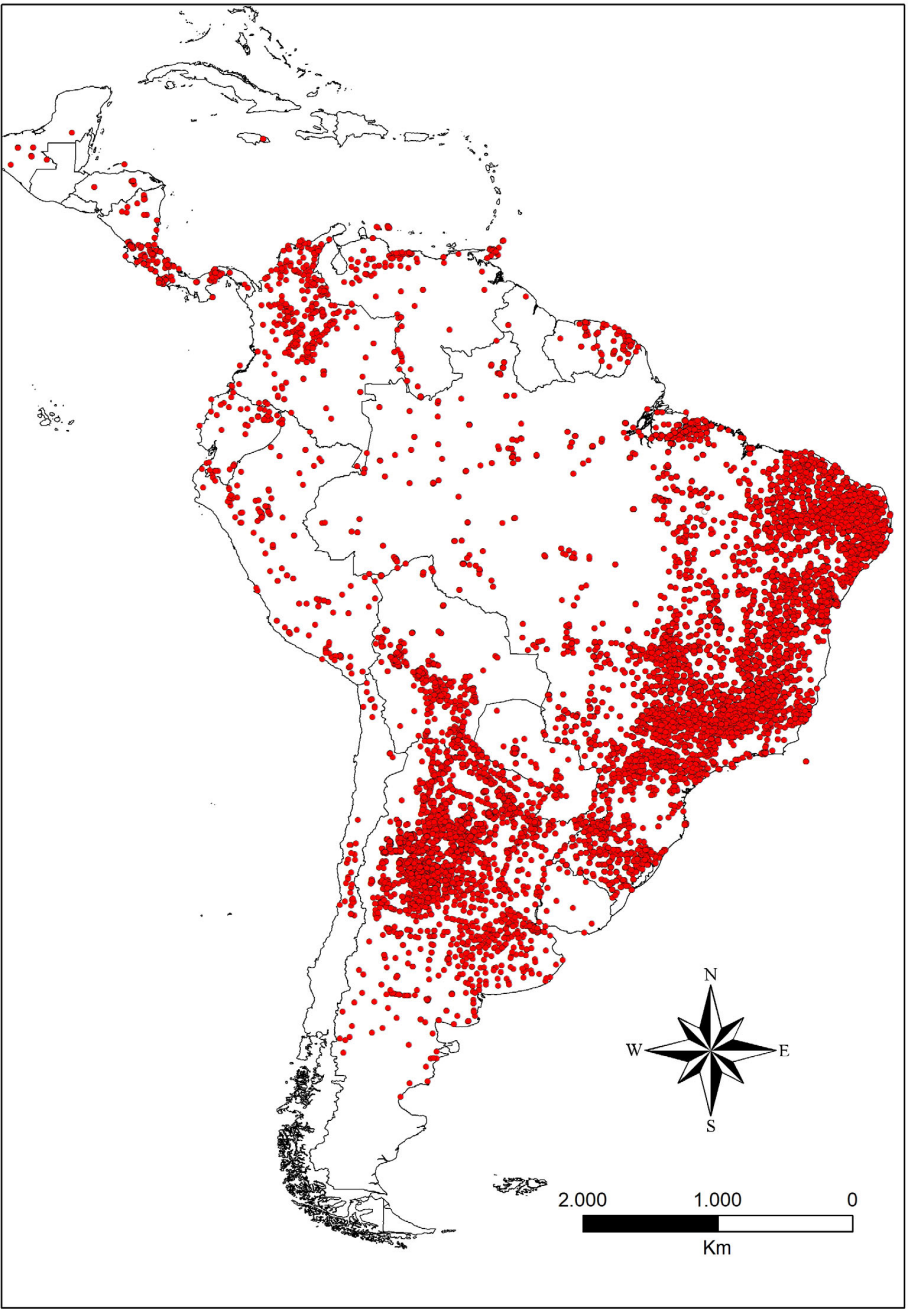
Geographical distribution of occurrence records of triatomine species used in the distribution model for both present and future climatic conditions.

We compiled occurrence records for 65 triatomine species as potential Chagas disease vectors in Brazil (Galvão & Gurgel‐Gonçalves, 2015; Jurberg et al., 2014). In total, 9359 primary records were obtained from the *Evandro Chagas Institute*, a leading research institution specialising in tropical medicine and vector‐borne diseases. These records, dating back to the 1950s, include historical entomological surveys, public reports of insect specimens submitted by the public for identification and data from multiple research projects conducted over more than 50 years. As such, all these records were taxonomically validated by specialists at the institute. Additionally, we compiled 17,780 secondary occurrence records from entomological collections available through digital biodiversity databases, including GBIF (https://www.gbif.org/), SpeciesLink (https://specieslink.net/) and Map of Life (https://mol.org/), as well as scientific literature searches in Web of Science (http://www.webofknowledge.com), Google Scholar (https://scholar.google.com.br/), Scopus (https://www.scopus.com) and the Scientific Electronic Library Online (SciELO, http://www.scielo.org), using species names as keywords. In total, we gathered 27,139 occurrence records of potential Chagas disease vectors dating back to the 1950s before applying data‐cleaning procedures. To reduce spatial autocorrelation and overfitting, we conducted a Moran's correlogram analysis (based on the linear distance between points) to identify and remove redundant, spatially autocorrelated and/or duplicate records. Our final dataset included 11,640 unique occurrence points across Central and South America (Figure 1; Table S1).

#### Species distribution modelling (SDM) procedures

We developed species distribution modelling (SDMs) for 55 species that met our minimum data criteria (Table S2), incorporating presence‐only occurrence points across the Neotropical region (Pimenta et al., 2022). The models were trained using current climate conditions (1970–2000; WorldClim version 2.1) and future climate projections for 2050 and 2080. We employed two climate scenarios: (i) SSP2‐4.5—an intermediate emissions scenario, where climate trends remain relatively stable compared to historical patterns; and (ii) SSP5‐8.5—a high‐emissions ‘fossil‐fuelled development’ scenario, characterised by rapid economic growth and high fossil fuel dependency. Climate projections were sourced from the Coupled Model Intercomparison Project Phase 6 (CMIP6; Eyring et al., 2016) and Global Climate Model (GCM; Christian et al., 2022). All modelling was based on Ecological Niche Modelling (ENM), following the approach described by Andrade et al. (2020).

#### Environmental variables

To minimise multicollinearity among environmental predictors, we conducted Principal Component Analysis (PCA) (Legendre & Legendre, 2012) on 19 bioclimatic variables from the WorldClim 2.1 database (http://www.worldclim.org/; Fick & Hijmans, 2017). The first seven principal components, which explained at least 95% of the variance, were retained for model training (Nobrega & De Marco, 2011). The bioclimatic variables used included a group of monthly climate variables sampled between 1970 and 2000, at a 9.4 × 9.4 km resolution, and which have been commonly used in Species Distribution Modelling (Lee et al., 2016).

To ensure the comparability of climate data across time and avoid methodological inconsistencies, we performed a PCA using only the present‐day climatic variables. The resulting loadings (i.e., eigenvectors) were then used to project future climate data from each selected AOGCM onto the same PCA space defined by the current climate. This procedure allows the scores for future conditions to be computed based on the same axes of climatic variation derived from the present, maintaining a consistent multivariate structure across time. By avoiding independent PCAs for each temporal scenario, we prevent distortions in variable space that would compromise model comparability and transferability (Sillero & Barbosa, 2021). This approach, known as PCA transfer, is crucial for the valid temporal projection of ecological niche models and has been successfully applied in recent studies involving climate change scenarios (Ferreira Leão et al., 2023).

### Modelling algorithms and ensemble approach

We employed four machine learning algorithms to predict species distributions: (i) Maxent (MXE) (Phillips et al., 2017), (ii) Random Forest (RDF) (Prasad et al., 2006), (iii) Support Vector Machine (SVM) (Guo et al., 2005) and (iv) Bayesian Gaussian (GAU) (Golding & Bolsa, 2016). To address uncertainties in individual model predictions, we used an ensemble approach, averaging suitability across models exceeding the mean thresholds for each species (Pimenta et al., 2022; Velazco et al., 2019). The Jaccard threshold was chosen to minimise omission and commission errors (Pimenta et al., 2022), and a binary occurrence map was generated with suitability values above the threshold indicating species presence. Spatial restrictions have been applied to reduce over‐prediction in distribution models (Mendes et al., 2020; Pimenta et al., 2022). Specifically, only pixels with species records or near predicted occurrence points were retained in the potential distribution map of the species (Mendes et al., 2020; Pimenta et al., 2022).

#### Model evaluation

To validate the model performance, we implemented two data partitioning strategies: a (i) a geographic partition structured as a checkerboard for species with more than 30 occurrence points; and (ii) a random bootstrap partition with 10 replicates, allocating 70% of the data for model training and 30% for testing for species with fewer than 30 occurrence points (Pimenta et al., 2022). Model Accuracy was assessed using Receiver Operating Characteristic (ROC) curves and the True Skill Statistic (TSS), which is widely recognised as an appropriate discrimination metric, independent of prevalence (Allouche et al., 2006; Shabani et al., 2018). TSS intuitively evaluates SDM performance by calculating the sensitivity (true positive rate, TPR) and specificity (true negative rate, TNR) based on presence–absence predictions. TSS values range from −1 (i.e., low predictive capacity) to +1 (better predictive performance and accuracy). Given the potential inflation of TSS values due to true negatives for species with low prevalence (Lawson et al., 2014), we focused evaluation metrics on true positive, false positive and false negative rates for a more balanced evaluation of model performance (Leroy et al., 2018).

### Data analysis

To assess potential shifts in species distribution, we mapped the species richness of potential Chagas disease vectors across Central and South America by summing the binary outputs of Species Distribution Modelling under present and future climate conditions. For each species, we compared current distributions (1970–2000 climate data) with projections for 2050 and 2080, using the SSP2‐4.5 (moderate warming) and SSP5‐8.5 (high emissions) scenarios. Using this approach, we classified areas based on changes in species' predicted distribution into three categories: (i) range contraction—areas where a species is present under current conditions but absent in future projections; (ii) range expansion—areas where a species is absent under current conditions but predicted to be present in the future; and (iii) stable distribution—areas where species presence remains unchanged across all timeframes. To aid in the identification of future hotspots for Chagas disease risk, we summed all maps to create richness maps for both scenarios for 2080.

## RESULTS

We conducted SDMs for 55 of the 65 species identified as Chagas disease vectors (Tables S1–S3). Ten species were excluded because they had fewer than five occurrence points, as species distribution modelling require a minimum sample size for reliable prediction. Among the 55 modelled species, unique occurrence points ranged from five records (*Triatoma jurbergi* Carcavallo, Galvão & Lent, and *Triatoma vandae* Carcavallo, Jurberg, Rocha, Galvão, Noireau & Lent) to 2170 records (*Triatoma infestans* (Klug)), reflecting variations in data availability and geographic spread. Current species richness patterns indicate high triatomine diversity in eastern South America, whereas lower richness was observed in the southern and northern regions (Figure S1).

**FIGURE 2:**
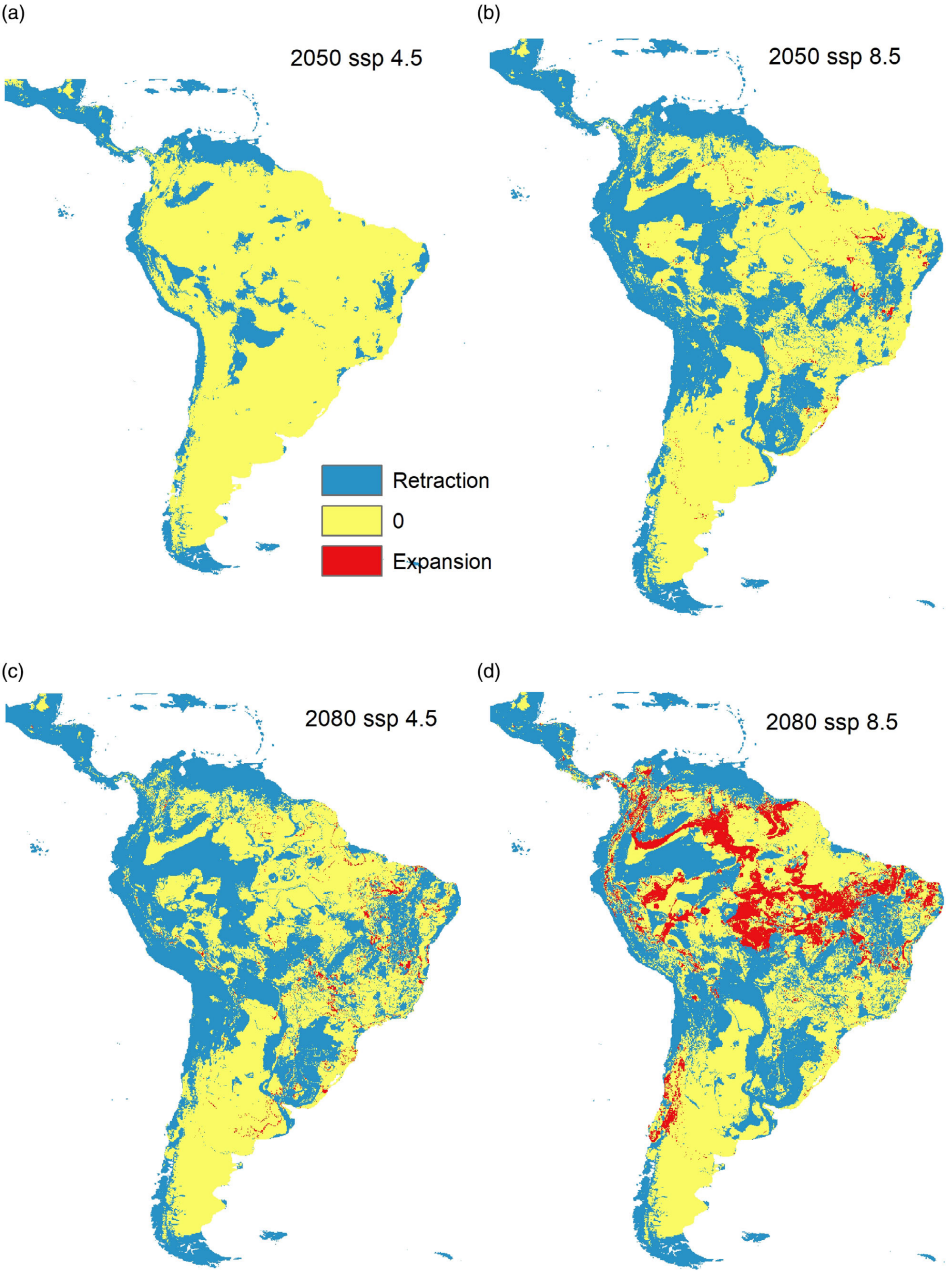
Effect of climate change on the retraction (blue areas) and expansion (red areas) of suitable climate distribution areas for triatomine species, comparing the present to the future in 2050 with mild changes (a), and the future in 2080 with significant changes (d).

Future projections followed a similar pattern but revealed notable diversity losses driven by shifts in many species in northern and western South America and near the Andean region, alongside significant expansion in central regions, particularly within the Brazilian Amazon (Figure 2). Under the SSP2–4.5 scenario, 4 species showed stable distributions, 27 exhibited range expansions and 24 experienced range contractions. Under the SSP5–8.5 scenario, the same four species remained stable, but 31 expanded their range, while 20 experienced range contraction (Table S2). In the near‐term projections (2050), the models indicated stability in suitable habitats under both climate scenarios. However, long‐term projections (2080) indicate substantial shifts in species distributions, particularly under the SSP5‐8.5 scenario, where an increase in suitability for the triatomine vector is projected in the Brazilian Amazon (Figure 2).

## DISCUSSION

Our results indicate that climate change is likely to significantly alter the distribution of potential vectors of Chagas disease in Brazil, particularly under extreme warming scenarios and towards the end of the century. Although our models for 2050 suggest relative stability in vector distributions, this trend may create a false sense of security, potentially leading to a decline in public health interventions. A similar pattern has been observed for other vector‐borne diseases in Brazil, such as yellow fever and measles, where reduced cases led to decreased preventive efforts, followed by resurgence (Oliveira et al., 2022). However, by 2080, our models predicted a significant expansion of vector ranges in the Brazilian Amazonia, suggesting an urgent need for long‐term disease surveillance and prevention.

Our results are in contrast with previous projections of declines in vector distribution in certain regions of South America by the mid‐century (Ceccarelli & Rabinovich, 2015; Medone et al., 2015). For example, Medone et al. (2015) found that areas currently considered to be at high to moderate risk of transmission by two vectors in Venezuela and Argentina are projected to reduce environmental suitability by 2050, whereas Ceccarelli and Rabinovich (2015) projected similar distribution trends for five species in Venezuela by 2060 and 2080. Several factors may explain these differences, including the improved climate projections. Our study employs updated climate change scenarios (CMIP6), which provide more refined temperature and precipitation projections than previous models available in 2015 (e.g., CMIP5). Furthermore, by modelling 55 species, our greater taxonomic coverage offers a broader view of potential distribution shifts in triatomine vectors. Nonetheless, as in Medone et al. (2015), our results do not indicate significant concerns regarding the expansion of vector distributions by 2050. However, concerns arise when examining our projected results for 2080, which are more alarming than the predictions of Ceccarelli and Rabinovich (2015).

One of the most concerning findings is the projected increase in vector suitability within the Brazilian Amazon. Our models suggest that while species may lose suitable habitats in the Brazilian Cerrado savannas and western South America (e.g., near the Andes), they will likely gain climatically suitable areas in the Amazonian frontiers, particularly in the deforestation arc. This region, characterised by expanding agricultural frontiers, illegal mining, cattle ranching and deforestation (Brando et al., 2013; Simmons, 2004), already faces land conflicts, weak governance and poor healthcare infrastructure (De Oliveira, 2008). Furthermore, a large portion of the population in this region lives in precarious housing and sanitary conditions (Sathler et al., 2018; Simmons, 2004), which increases the likelihood of human‐vector contact (Jurberg et al., 2014).

Deforestation and climate change are projected to enhance temperatures in the Brazilian Amazonia (Oliveira et al., 2021), which could further impact Chagas disease transmission by altering parasite–vector interactions (Loshouarn & Guarneri, 2024), particularly in peri‐urban and urban areas. Higher temperatures may also accelerate the infectious cycle of protozoans and increase their transmission rates (Elliot et al., 2015; Tamayo et al., 2018). Additionally, changes in climate could shift the distribution of mammalian reservoirs of *T. cruzi* in the Amazon region (Morueta‐Holme et al., 2010; Ribeiro et al., 2016), further influencing vector–host dynamics. These factors highlight the importance of looking at human–nature interactions—including the role of socio‐environmental factors (Prist et al., 2016, 2023)—to understand the landscape ecology of *T. cruzi* (López‐Cancino et al., 2015).

Climate change also intensifies extreme weather events, which may further disrupt the vector ecology. For example, the El Niño event of 2023 contributed to record‐breaking temperatures in Brazil (INMET, 2023), causing widespread flooding in the south (Reboita et al., 2024) and an unprecedented drought in Amazonia (Marengo et al., 2024). This extreme heat event resulted in the mass mortality of aquatic mammals, including 100 Amazonian River dolphins (Ionova & Albeck‐Ripka, 2023). While our study focused on long‐term climate trends, these findings highlight how future scenarios may be compounded by increased climatic variability, potentially making vector species distribution even less predictable. Collectively, our results underscore the urgent need for continued vector surveillance and climate‐adaptive public health strategies. By identifying areas at risk for future vector expansion, our findings can support early intervention strategies to mitigate potential disease outbreaks.

## AUTHOR CONTRIBUTIONS

**Leandro Schlemmer Brasil:** Conceptualization; writing – review and editing; writing – original draft; data curation. **Divino Vicente Silvério:** Writing – review and editing. **José Orlando de Almeida Silva:** Writing – original draft; writing – review and editing. **Walter Souza Santos:** Visualization; validation. **Leonardo Viana de Melo:** Writing – review and editing. **Leandro Juen:** Writing – review and editing. **Filipe Machado França:** Writing – review and editing. **Thiago Bernardi Vieira:** Writing – review and editing; formal analysis.

## CONFLICT OF INTEREST STATEMENT

The authors declare no conflicts of interest.

## ETHICS STATEMENT

We do not conduct human studies, animal studies, clinical trial registration and biosafety studies.

## Supporting information


**Table S1.** Locations sampled between 2017 and 2022. Points are characterized by the state, municipality, geographic coordinates (Longitude—Long, and Latitude—Lat), date and sampled method (C—Bat Colony, H—Ran Over and M—Mist Net).
**Table S2**. List of species and their status of unevaluated, retraction or movement.
**Table S3**. Summary of threshold, AUC, Kappa, TSS, and Jaccard values for the algorithm of each species and for the assembly of species (SUP).
**Figure S1**. Richness distribution model for triatomine species richness of triatomines in the present (A), moderate‐warming scenario (SSP2‐4.5) future in 2050 with mild changes (B), high‐emission scenariofuture in 2050 (SSP5‐8.5) with significant changes (C), moderate‐warming scenario (SSP2‐4.5) future in 2080 with mild changes (D), high‐emission scenarioand future in 2080 with significant changes (E).

## Data Availability

All data are provided in the Supporting Information. GBIF data can be found at https://doi.org/10.15468/dl.2wudy4.
