# Supplementary material for: Potential geographic displacement of Chagas disease vectors under climate change
Source: Med Vet Entomol. 2025 May 21;39(4):709–17. doi: 10.1111/mve.12810 (PMC12586274; doi:10.1111/mve.12810)
Supplement: Supplementary file 1 — Table S1. Locations sampled between 2017 and 2022. Points are characterized by the state, municipality, geographic coordinates (Longitude—Long, and Latitude—Lat), date and sampled method (C—Bat Colony, H—Ran Over and M—Mist Net). Table S2. List of species and their status of unevaluated, retraction or movement. Table S3. Summary of threshold, AUC, Kappa, TSS, and Jaccard values for the algorithm of each species and for the assembly of species (SUP). Figure S1. Richness distribution model for triatomine species richness of triatomines in the present (A), moderate‐warming scenario (SSP2‐4.5) future in 2050 with mild changes (B), high‐emission scenariofuture in 2050 (SSP5‐8.5) with significant changes (C), moderate‐warming scenario (SSP2‐4.5) future in 2080 with mild changes (D), high‐emission scenarioand future in 2080 with significant changes (E). [file MVE-39-709-s001.docx]

**Supporting Information**

**Table S1.** Locations sampled between 2017 and 2022. Points are characterized by the state, municipality, geographic coordinates (Longitude – Long, and Latitude – Lat), date and sampled method (C – Bat Colony, H – Ran Over, and M – Mist Net).

<https://docs.google.com/spreadsheets/d/12zTxLmkkjYVc0kZiB9awQiqaOsaKe1lI/edit?usp=sharing&ouid=115978702443000936665&rtpof=true&sd=true>

**Table S2.** List of species and their status of unevaluated, retraction, or movement.

| Species | Occurrences | Projections 2080 | |
| --- | --- | --- | --- |
|  |  | ssp45 | ssp85 |
| *Alberprosenia malheiroi* | 2 | Not evaluated | Not evaluated |
| *Belminus laportei* | 6 | Retracts | Retracts |
| *Cavernicola lenti* | 4 | Not evaluated | Not evaluated |
| *Cavernicola pilosa* | 48 | Retracts | Move |
| *Eratyrus mucronatus* | 138 | Retracts | Move |
| *Microtriatoma borbai* | 6 | Move | Move |
| *Microtriatoma trinidadensis* | 23 | Move | Move |
| *Panstrongylus diasi* | 66 | Retracts | Move |
| *Panstrongylus geniculatus* | 827 | Retracts | Retracts |
| *Panstrongylus guentheri* | 106 | Retracts | Move |
| *Panstrongylus lenti* | 4 | Not evaluated | Not evaluated |
| *Panstrongylus lignarius* | 126 | Retracts | Expande |
| *Panstrongylus lutzi* | 464 | Retracts | Move |
| *Panstrongylus lutzii* | 2 | Not evaluated | Not evaluated |
| *Panstrongylus megistus* | 2105 | Move | Move |
| *Panstrongylus rufotuberculatus* | 154 | Retracts | Retracts |
| *Panstrongylus tupynambai* | 14 | Move | Move |
| *Parabelminus carioca* | 1 | Not evaluated | Not evaluated |
| *Parabelminus yurupucu* | 2 | Not evaluated | Not evaluated |
| *Psammolestes coreodes* | 3 | Not evaluated | Not evaluated |
| *Psammolestes tertius* | 86 | Move | Move |
| *Rhodnius amazonicus* | 8 | Move | Move |
| *Rhodnius brethesi* | 22 | Igual | Igual |
| *Rhodnius coreodes* | 114 | Move | Retracts |
| *Rhodnius domesticus* | 16 | Retracts | Retracts |
| *Rhodnius milesi* | 3 | Not evaluated | Not evaluated |
| *Rhodnius millesi* | 2 | Not evaluated | Not evaluated |
| *Rhodnius montenegrensis* | 17 | Retracts | Retracts |
| *Rhodnius nasutus* | 88 | Move | Retracts |
| *Rhodnius neglectus* | 168 | Igual | Igual |
| *Rhodnius paraensis* | 9 | Move | Move |
| *Rhodnius pictipes* | 265 | Move | Move |
| *Rhodnius robustus* | 200 | Retracts | Retracts |
| *Rhodnius stali* | 14 | Retracts | Retracts |
| *Rhodnius tertius* | 96 | Retracts | Move |
| *Triatoma arthurneivai* | 9 | Move | Move |
| *Triatoma baratai* | 11 | Move | Move |
| *Triatoma brasiliensis* | 825 | Retracts | Move |
| *Triatoma brasiliensis macromelasoma* | 1 | Not evaluated | Not evaluated |
| *Triatoma carcavalloi* | 12 | Move | Move |
| *Triatoma circummaculatus* | 23 | Move | Move |
| *Triatoma costalimai* | 43 | Retracts | Retracts |
| *Triatoma deaneorum* | 2 | Not evaluated | Not evaluated |
| *Triatoma delpontei* | 55 | Move | Move |
| *Triatoma guazu* | 6 | Move | Move |
| *Triatoma infestans* | 2170 | Move | Move |
| *Triatoma juazeirensis* | 18 | Retracts | Retracts |
| *Triatoma jurbergi* | 5 | Retracts | Retracts |
| *Triatoma klugi* | 4 | Not evaluated | Not evaluated |
| *Triatoma lenti* | 18 | Move | Move |
| *Triatoma maculata* | 158 | Retracts | Retracts |
| *Triatoma matogrossensis* | 13 | Retracts | Retracts |
| *Triatoma melanica* | 9 | Move | Move |
| *Triatoma melanocephala* | 112 | Move | Move |
| *Triatoma oliveirai* | 6 | Move | Move |
| *Triatoma petrochiae* | 35 | Move | Move |
| *Triatoma pintodiasi* | 1 | Not evaluated | Not evaluated |
| *Triatoma platensis* | 218 | Igual | Igual |
| *Triatoma pseudomaculata* | 1162 | Move | Move |
| *Triatoma rubrofasciata* | 24 | Retracts | Retracts |
| *Triatoma rubrovaria* | 121 | Igual | Igual |
| *Triatoma sherlocki* | 9 | Move | Move |
| *Triatoma sordida* | 1133 | Retracts | Retracts |
| *Triatoma tibiamaculata* | 46 | Retracts | Retracts |
| *Triatoma vandae* | 5 | Move | Move |
| *Triatoma vitticeps* | 151 | Move | Move |
| *Triatoma williami* | 24 | Move | Retracts |
| *Triatoma wygodzinskyi* | 9 | Retracts | Retracts |

**Table S3.** Summary of threshold, AUC, Kappa, TSS, and Jaccard values for the algorithm of each species and for the assembly of species (SUP).

| **Species** | **Algorithm** | **Partition** | **Threshold** | **AUC** | **Kappa** | **TSS** | **Jaccard** |
| --- | --- | --- | --- | --- | --- | --- | --- |
| *Belminus laportei* | GAU | BOOT | JACCARD | 1 | 1 | 1 | 1 |
| *Belminus laportei* | MXD | BOOT | JACCARD | 0.625 | 0.5 | 0.5 | 0.666666667 |
| *Belminus laportei* | RDF | BOOT | JACCARD | 0.7 | 0.55 | 0.55 | 0.7 |
| *Belminus laportei* | SUP | BOOT | JACCARD | 1 | 1 | 1 | 1 |
| *Belminus laportei* | SVM | BOOT | JACCARD | 1 | 1 | 1 | 1 |
| *Cavernicola pilosa* | MXD | BLOCK | JACCARD | 0.646942863 | 0.28584392 | 0.28584392 | 0.586645963 |
| *Cavernicola pilosa* | SVM | BLOCK | JACCARD | 0.808294439 | 0.548094374 | 0.548094374 | 0.66194332 |
| *Cavernicola pilosa* | RDF | BLOCK | JACCARD | 0.686939437 | 0.191470054 | 0.191470054 | 0.548642534 |
| *Cavernicola pilosa* | GAU | BLOCK | JACCARD | 0.744732395 | 0.426497278 | 0.426497278 | 0.607692308 |
| *Cavernicola pilosa* | SUP | BLOCK | JACCARD | 0.778574511 | 0.409255898 | 0.409255898 | 0.614906832 |
| *Eratyrus mucronatus* | MXD | BLOCK | JACCARD | 0.76471322 | 0.492662233 | 0.492662233 | 0.655971381 |
| *Eratyrus mucronatus* | SVM | BLOCK | JACCARD | 0.777593695 | 0.491057097 | 0.491057097 | 0.644140099 |
| *Eratyrus mucronatus* | RDF | BLOCK | JACCARD | 0.768400796 | 0.472712681 | 0.472712681 | 0.643789474 |
| *Eratyrus mucronatus* | GAU | BLOCK | JACCARD | 0.763415866 | 0.462508599 | 0.462508599 | 0.632521645 |
| *Eratyrus mucronatus* | SUP | BLOCK | JACCARD | 0.791849002 | 0.540128411 | 0.540128411 | 0.667162698 |
| *Microtriatoma borbai* | GAU | BOOT | JACCARD | 0.375 | 0.05 | 0.05 | 0.516666667 |
| *Microtriatoma borbai* | MXD | BOOT | JACCARD | 0.95 | 0.9 | 0.9 | 0.933333333 |
| *Microtriatoma borbai* | RDF | BOOT | JACCARD | 0.65 | 0.35 | 0.35 | 0.633333333 |
| *Microtriatoma borbai* | SUP | BOOT | JACCARD | 0.925 | 0.85 | 0.85 | 0.9 |
| *Microtriatoma borbai* | SVM | BOOT | JACCARD | 0.475 | 0.1 | 0.1 | 0.533333333 |
| *Microtriatoma trinidadensis* | GAU | BOOT | JACCARD | 0.795918367 | 0.528571429 | 0.528571429 | 0.6629329 |
| *Microtriatoma trinidadensis* | MXD | BOOT | JACCARD | 0.897959184 | 0.7 | 0.7 | 0.763888889 |
| *Microtriatoma trinidadensis* | RDF | BOOT | JACCARD | 0.828571429 | 0.671428571 | 0.671428571 | 0.737973138 |
| *Microtriatoma trinidadensis* | SUP | BOOT | JACCARD | 0.857142857 | 0.671428571 | 0.671428571 | 0.740025253 |
| *Microtriatoma trinidadensis* | SVM | BOOT | JACCARD | 0.746938776 | 0.5 | 0.5 | 0.630021645 |
| *Panstrongylus diasi* | MXD | BLOCK | JACCARD | 0.933576854 | 0.786764706 | 0.786764706 | 0.822435897 |
| *Panstrongylus diasi* | SVM | BLOCK | JACCARD | 0.887538184 | 0.709558824 | 0.709558824 | 0.748094248 |
| *Panstrongylus diasi* | RDF | BLOCK | JACCARD | 0.863162981 | 0.681985294 | 0.681985294 | 0.717532468 |
| *Panstrongylus diasi* | GAU | BLOCK | JACCARD | 0.89309175 | 0.738970588 | 0.738970588 | 0.768393393 |
| *Panstrongylus diasi* | SUP | BLOCK | JACCARD | 0.907153912 | 0.771139706 | 0.771139706 | 0.78869969 |
| *Panstrongylus geniculatus* | MXD | BLOCK | JACCARD | 0.663860524 | 0.164577114 | 0.164577114 | 0.542128194 |
| *Panstrongylus geniculatus* | SVM | BLOCK | JACCARD | 0.678327602 | 0.189853673 | 0.189853673 | 0.522194592 |
| *Panstrongylus geniculatus* | RDF | BLOCK | JACCARD | 0.702239562 | 0.257723149 | 0.257723149 | 0.558964028 |
| *Panstrongylus geniculatus* | GAU | BLOCK | JACCARD | 0.672674004 | 0.171837869 | 0.171837869 | 0.53803385 |
| *Panstrongylus geniculatus* | SUP | BLOCK | JACCARD | 0.702239562 | 0.257723149 | 0.257723149 | 0.558964028 |
| *Panstrongylus guentheri* | MXD | BLOCK | JACCARD | 0.933539734 | 0.800035804 | 0.800035804 | 0.8261345 |
| *Panstrongylus guentheri* | SVM | BLOCK | JACCARD | 0.909002661 | 0.760651629 | 0.760651629 | 0.801159274 |
| *Panstrongylus guentheri* | RDF | BLOCK | JACCARD | 0.893547869 | 0.759219477 | 0.759219477 | 0.803447421 |
| *Panstrongylus guentheri* | GAU | BLOCK | JACCARD | 0.895976066 | 0.781059792 | 0.781059792 | 0.812367585 |
| *Panstrongylus guentheri* | SUP | BLOCK | JACCARD | 0.922476451 | 0.781059792 | 0.781059792 | 0.812367585 |
| *Panstrongylus lignarius* | MXD | BLOCK | JACCARD | 0.734638804 | 0.292207792 | 0.292207792 | 0.554632867 |
| *Panstrongylus lignarius* | SVM | BLOCK | JACCARD | 0.790416183 | 0.519480519 | 0.519480519 | 0.672925457 |
| *Panstrongylus lignarius* | RDF | BLOCK | JACCARD | 0.818388344 | 0.59554731 | 0.59554731 | 0.69109241 |
| *Panstrongylus lignarius* | GAU | BLOCK | JACCARD | 0.830385755 | 0.574211503 | 0.574211503 | 0.692942943 |
| *Panstrongylus lignarius* | SUP | BLOCK | JACCARD | 0.819338361 | 0.560296846 | 0.560296846 | 0.690497076 |
| *Panstrongylus lutzi* | MXD | BLOCK | JACCARD | 0.964724229 | 0.861588932 | 0.861588932 | 0.875925926 |
| *Panstrongylus lutzi* | SVM | BLOCK | JACCARD | 0.967652391 | 0.86685209 | 0.86685209 | 0.881415506 |
| *Panstrongylus lutzi* | RDF | BLOCK | JACCARD | 0.956167105 | 0.85497076 | 0.85497076 | 0.868942834 |
| *Panstrongylus lutzi* | GAU | BLOCK | JACCARD | 0.961749324 | 0.86685209 | 0.86685209 | 0.880155298 |
| *Panstrongylus lutzi* | SUP | BLOCK | JACCARD | 0.966074149 | 0.873470261 | 0.873470261 | 0.885421785 |
| *Panstrongylus megistus* | MXD | BLOCK | JACCARD | 0.91636086 | 0.731173964 | 0.731173964 | 0.780013263 |
| *Panstrongylus megistus* | SVM | BLOCK | JACCARD | 0.913421549 | 0.709243292 | 0.709243292 | 0.766768557 |
| *Panstrongylus megistus* | RDF | BLOCK | JACCARD | 0.910130657 | 0.728181756 | 0.728181756 | 0.779455827 |
| *Panstrongylus megistus* | GAU | BLOCK | JACCARD | 0.915393044 | 0.749381872 | 0.749381872 | 0.792476305 |
| *Panstrongylus megistus* | SUP | BLOCK | JACCARD | 0.919580042 | 0.753738116 | 0.753738116 | 0.7965389 |
| *Panstrongylus rufotuberculatus* | MXD | BLOCK | JACCARD | 0.720357565 | 0.214285714 | 0.214285714 | 0.552506909 |
| *Panstrongylus rufotuberculatus* | SVM | BLOCK | JACCARD | 0.847613426 | 0.538961039 | 0.538961039 | 0.656596029 |
| *Panstrongylus rufotuberculatus* | RDF | BLOCK | JACCARD | 0.832518131 | 0.545454545 | 0.545454545 | 0.653972195 |
| *Panstrongylus rufotuberculatus* | GAU | BLOCK | JACCARD | 0.836903356 | 0.590909091 | 0.590909091 | 0.691047485 |
| *Panstrongylus rufotuberculatus* | SUP | BLOCK | JACCARD | 0.849384382 | 0.545454545 | 0.545454545 | 0.662324176 |
| *Panstrongylus tupynambai* | GAU | BOOT | JACCARD | 0.9875 | 0.975 | 0.975 | 0.975 |
| *Panstrongylus tupynambai* | MXD | BOOT | JACCARD | 1 | 1 | 1 | 1 |
| *Panstrongylus tupynambai* | RDF | BOOT | JACCARD | 1 | 1 | 1 | 1 |
| *Panstrongylus tupynambai* | SUP | BOOT | JACCARD | 1 | 1 | 1 | 1 |
| *Panstrongylus tupynambai* | SVM | BOOT | JACCARD | 0.875 | 0.8 | 0.8 | 0.9 |
| *Psammolestes tertius* | MXD | BLOCK | JACCARD | 0.936902654 | 0.837662338 | 0.837662338 | 0.854166667 |
| *Psammolestes tertius* | SVM | BLOCK | JACCARD | 0.931623751 | 0.850108225 | 0.850108225 | 0.863718821 |
| *Psammolestes tertius* | RDF | BLOCK | JACCARD | 0.915618382 | 0.780844156 | 0.780844156 | 0.810691824 |
| *Psammolestes tertius* | GAU | BLOCK | JACCARD | 0.930641058 | 0.804112554 | 0.804112554 | 0.833600963 |
| *Psammolestes tertius* | SUP | BLOCK | JACCARD | 0.939106979 | 0.861471861 | 0.861471861 | 0.872222222 |
| *Rhodnius amazonicus* | GAU | BOOT | JACCARD | 1 | 1 | 1 | 1 |
| *Rhodnius amazonicus* | MXD | BOOT | JACCARD | 1 | 1 | 1 | 1 |
| *Rhodnius amazonicus* | RDF | BOOT | JACCARD | 1 | 1 | 1 | 1 |
| *Rhodnius amazonicus* | SUP | BOOT | JACCARD | 1 | 1 | 1 | 1 |
| *Rhodnius amazonicus* | SVM | BOOT | JACCARD | 1 | 1 | 1 | 1 |
| *Rhodnius brethesi* | GAU | BOOT | JACCARD | 0.955102041 | 0.914285714 | 0.914285714 | 0.914285714 |
| *Rhodnius brethesi* | MXD | BOOT | JACCARD | 0.971428571 | 0.9 | 0.9 | 0.913492063 |
| *Rhodnius brethesi* | RDF | BOOT | JACCARD | 0.944897959 | 0.885714286 | 0.885714286 | 0.894642857 |
| *Rhodnius brethesi* | SUP | BOOT | JACCARD | 0.96122449 | 0.928571429 | 0.928571429 | 0.928571429 |
| *Rhodnius brethesi* | SVM | BOOT | JACCARD | 0.944897959 | 0.914285714 | 0.914285714 | 0.916071429 |
| *Rhodnius coreodes* | MXD | BLOCK | JACCARD | 0.960056132 | 0.824199507 | 0.824199507 | 0.845238095 |
| *Rhodnius coreodes* | SVM | BLOCK | JACCARD | 0.977518943 | 0.868534483 | 0.868534483 | 0.883838384 |
| *Rhodnius coreodes* | RDF | BLOCK | JACCARD | 0.941234394 | 0.778325123 | 0.778325123 | 0.828738513 |
| *Rhodnius coreodes* | GAU | BLOCK | JACCARD | 0.973905882 | 0.877463054 | 0.877463054 | 0.886039133 |
| *Rhodnius coreodes* | SUP | BLOCK | JACCARD | 0.976689331 | 0.868534483 | 0.868534483 | 0.878870674 |
| *Rhodnius domesticus* | GAU | BOOT | JACCARD | 0.748 | 0.56 | 0.56 | 0.644722222 |
| *Rhodnius domesticus* | MXD | BOOT | JACCARD | 0.796 | 0.6 | 0.6 | 0.67297619 |
| *Rhodnius domesticus* | RDF | BOOT | JACCARD | 0.794 | 0.58 | 0.58 | 0.681865079 |
| *Rhodnius domesticus* | SUP | BOOT | JACCARD | 0.824 | 0.7 | 0.7 | 0.7425 |
| *Rhodnius domesticus* | SVM | BOOT | JACCARD | 0.784 | 0.64 | 0.64 | 0.716150794 |
| *Rhodnius montenegrensis* | GAU | BOOT | JACCARD | 0.916 | 0.86 | 0.86 | 0.87 |
| *Rhodnius montenegrensis* | MXD | BOOT | JACCARD | 0.872 | 0.7 | 0.7 | 0.744761905 |
| *Rhodnius montenegrensis* | RDF | BOOT | JACCARD | 0.836 | 0.64 | 0.64 | 0.724761905 |
| *Rhodnius montenegrensis* | SUP | BOOT | JACCARD | 0.896 | 0.74 | 0.74 | 0.8 |
| *Rhodnius montenegrensis* | SVM | BOOT | JACCARD | 0.872 | 0.72 | 0.72 | 0.783333333 |
| *Rhodnius nasutus* | MXD | BLOCK | JACCARD | 0.917400519 | 0.769117647 | 0.769117647 | 0.789312039 |
| *Rhodnius nasutus* | SVM | BLOCK | JACCARD | 0.84275519 | 0.663235294 | 0.663235294 | 0.732854495 |
| *Rhodnius nasutus* | RDF | BLOCK | JACCARD | 0.868516436 | 0.688235294 | 0.688235294 | 0.74746988 |
| *Rhodnius nasutus* | GAU | BLOCK | JACCARD | 0.885320069 | 0.733823529 | 0.733823529 | 0.790208016 |
| *Rhodnius nasutus* | SUP | BLOCK | JACCARD | 0.894394464 | 0.726470588 | 0.726470588 | 0.782407407 |
| *Rhodnius neglectus* | MXD | BLOCK | JACCARD | 0.878402129 | 0.645071825 | 0.645071825 | 0.729722964 |
| *Rhodnius neglectus* | SVM | BLOCK | JACCARD | 0.87867471 | 0.620466505 | 0.620466505 | 0.697928967 |
| *Rhodnius neglectus* | RDF | BLOCK | JACCARD | 0.894263364 | 0.655596643 | 0.655596643 | 0.705569728 |
| *Rhodnius neglectus* | GAU | BLOCK | JACCARD | 0.883032536 | 0.692149054 | 0.692149054 | 0.731414613 |
| *Rhodnius neglectus* | SUP | BLOCK | JACCARD | 0.898844615 | 0.697767032 | 0.697767032 | 0.734373296 |
| *Rhodnius paraensis* | GAU | BOOT | JACCARD | 0.977777778 | 0.933333333 | 0.933333333 | 0.95 |
| *Rhodnius paraensis* | MXD | BOOT | JACCARD | 1 | 1 | 1 | 1 |
| *Rhodnius paraensis* | RDF | BOOT | JACCARD | 0.977777778 | 0.933333333 | 0.933333333 | 0.95 |
| *Rhodnius paraensis* | SUP | BOOT | JACCARD | 1 | 1 | 1 | 1 |
| *Rhodnius paraensis* | SVM | BOOT | JACCARD | 0.988888889 | 0.966666667 | 0.966666667 | 0.975 |
| *Rhodnius pictipes* | MXD | BLOCK | JACCARD | 0.846467395 | 0.56253192 | 0.56253192 | 0.664519951 |
| *Rhodnius pictipes* | SVM | BLOCK | JACCARD | 0.829248792 | 0.551295965 | 0.551295965 | 0.663863727 |
| *Rhodnius pictipes* | RDF | BLOCK | JACCARD | 0.851686792 | 0.522823034 | 0.522823034 | 0.663325886 |
| *Rhodnius pictipes* | GAU | BLOCK | JACCARD | 0.842413344 | 0.537282942 | 0.537282942 | 0.669807853 |
| *Rhodnius pictipes* | SUP | BLOCK | JACCARD | 0.856097399 | 0.525855465 | 0.525855465 | 0.664672014 |
| *Rhodnius robustus* | MXD | BLOCK | JACCARD | 0.804443583 | 0.518051665 | 0.518051665 | 0.664393939 |
| *Rhodnius robustus* | SVM | BLOCK | JACCARD | 0.820829303 | 0.535377114 | 0.535377114 | 0.665 |
| *Rhodnius robustus* | RDF | BLOCK | JACCARD | 0.834974254 | 0.551924473 | 0.551924473 | 0.681818182 |
| *Rhodnius robustus* | GAU | BLOCK | JACCARD | 0.836352836 | 0.582788671 | 0.582788671 | 0.695532915 |
| *Rhodnius robustus* | SUP | BLOCK | JACCARD | 0.839513804 | 0.559809109 | 0.559809109 | 0.682016587 |
| *Rhodnius stali* | GAU | BOOT | JACCARD | 1 | 1 | 1 | 1 |
| *Rhodnius stali* | MXD | BOOT | JACCARD | 0.9125 | 0.875 | 0.875 | 0.885 |
| *Rhodnius stali* | RDF | BOOT | JACCARD | 1 | 1 | 1 | 1 |
| *Rhodnius stali* | SUP | BOOT | JACCARD | 1 | 1 | 1 | 1 |
| *Rhodnius stali* | SVM | BOOT | JACCARD | 1 | 0.95 | 0.95 | 0.95 |
| *Rhodnius tertius* | MXD | BLOCK | JACCARD | 0.905058896 | 0.705133831 | 0.705133831 | 0.735472371 |
| *Rhodnius tertius* | SVM | BLOCK | JACCARD | 0.846678402 | 0.668933743 | 0.668933743 | 0.729058788 |
| *Rhodnius tertius* | RDF | BLOCK | JACCARD | 0.854120197 | 0.605748135 | 0.605748135 | 0.691758242 |
| *Rhodnius tertius* | GAU | BLOCK | JACCARD | 0.857204427 | 0.565818341 | 0.565818341 | 0.664520548 |
| *Rhodnius tertius* | SUP | BLOCK | JACCARD | 0.880980858 | 0.685607723 | 0.685607723 | 0.747826087 |
| *Triatoma arthurneivai* | GAU | BOOT | JACCARD | 1 | 1 | 1 | 1 |
| *Triatoma arthurneivai* | MXD | BOOT | JACCARD | 1 | 1 | 1 | 1 |
| *Triatoma arthurneivai* | RDF | BOOT | JACCARD | 1 | 1 | 1 | 1 |
| *Triatoma arthurneivai* | SUP | BOOT | JACCARD | 1 | 1 | 1 | 1 |
| *Triatoma arthurneivai* | SVM | BOOT | JACCARD | 1 | 1 | 1 | 1 |
| *Triatoma baratai* | GAU | BOOT | JACCARD | 1 | 1 | 1 | 1 |
| *Triatoma baratai* | MXD | BOOT | JACCARD | 1 | 1 | 1 | 1 |
| *Triatoma baratai* | RDF | BOOT | JACCARD | 0.977777778 | 0.966666667 | 0.966666667 | 0.975 |
| *Triatoma baratai* | SUP | BOOT | JACCARD | 1 | 1 | 1 | 1 |
| *Triatoma baratai* | SVM | BOOT | JACCARD | 1 | 1 | 1 | 1 |
| *Triatoma brasiliensis* | MXD | BLOCK | JACCARD | 0.949622393 | 0.79063004 | 0.79063004 | 0.822941879 |
| *Triatoma brasiliensis* | SVM | BLOCK | JACCARD | 0.941149616 | 0.817405031 | 0.817405031 | 0.838431174 |
| *Triatoma brasiliensis* | RDF | BLOCK | JACCARD | 0.941555747 | 0.81170001 | 0.81170001 | 0.834303504 |
| *Triatoma brasiliensis* | GAU | BLOCK | JACCARD | 0.95007385 | 0.838891081 | 0.838891081 | 0.856121454 |
| *Triatoma brasiliensis* | SUP | BLOCK | JACCARD | 0.948732872 | 0.837771525 | 0.837771525 | 0.854427309 |
| *Triatoma carcavalloi* | GAU | BOOT | JACCARD | 1 | 1 | 1 | 1 |
| *Triatoma carcavalloi* | MXD | BOOT | JACCARD | 1 | 1 | 1 | 1 |
| *Triatoma carcavalloi* | RDF | BOOT | JACCARD | 0.99375 | 0.95 | 0.95 | 0.96 |
| *Triatoma carcavalloi* | SUP | BOOT | JACCARD | 1 | 1 | 1 | 1 |
| *Triatoma carcavalloi* | SVM | BOOT | JACCARD | 1 | 1 | 1 | 1 |
| *Triatoma circummaculatus* | GAU | BOOT | JACCARD | 1 | 1 | 1 | 1 |
| *Triatoma circummaculatus* | MXD | BOOT | JACCARD | 1 | 1 | 1 | 1 |
| *Triatoma circummaculatus* | RDF | BOOT | JACCARD | 1 | 1 | 1 | 1 |
| *Triatoma circummaculatus* | SUP | BOOT | JACCARD | 1 | 1 | 1 | 1 |
| *Triatoma circummaculatus* | SVM | BOOT | JACCARD | 1 | 1 | 1 | 1 |
| *Triatoma costalimai* | MXD | BLOCK | JACCARD | 0.842900333 | 0.617117117 | 0.617117117 | 0.706199461 |
| *Triatoma costalimai* | SVM | BLOCK | JACCARD | 0.833008684 | 0.738738739 | 0.738738739 | 0.777597403 |
| *Triatoma costalimai* | RDF | BLOCK | JACCARD | 0.817313936 | 0.684684685 | 0.684684685 | 0.740121581 |
| *Triatoma costalimai* | GAU | BLOCK | JACCARD | 0.785153397 | 0.657657658 | 0.657657658 | 0.724489796 |
| *Triatoma costalimai* | SUP | BLOCK | JACCARD | 0.828625923 | 0.725225225 | 0.725225225 | 0.766233766 |
| *Triatoma delpontei* | MXD | BLOCK | JACCARD | 0.887864352 | 0.705108359 | 0.705108359 | 0.769565217 |
| *Triatoma delpontei* | SVM | BLOCK | JACCARD | 0.845993204 | 0.59752322 | 0.59752322 | 0.732142857 |
| *Triatoma delpontei* | RDF | BLOCK | JACCARD | 0.851530974 | 0.715170279 | 0.715170279 | 0.744360902 |
| *Triatoma delpontei* | GAU | BLOCK | JACCARD | 0.84564934 | 0.702012384 | 0.702012384 | 0.751937984 |
| *Triatoma delpontei* | SUP | BLOCK | JACCARD | 0.857416203 | 0.731424149 | 0.731424149 | 0.768604651 |
| *Triatoma guazu* | GAU | BOOT | JACCARD | 0.275 | 0.1 | 0.1 | 0.533333333 |
| *Triatoma guazu* | MXD | BOOT | JACCARD | 0.375 | 0.25 | 0.25 | 0.583333333 |
| *Triatoma guazu* | RDF | BOOT | JACCARD | 0.225 | 0 | 0 | 0.5 |
| *Triatoma guazu* | SUP | BOOT | JACCARD | 0.475 | 0.35 | 0.35 | 0.633333333 |
| *Triatoma guazu* | SVM | BOOT | JACCARD | 0.475 | 0.25 | 0.25 | 0.6 |
| *Triatoma infestans* | MXD | BLOCK | JACCARD | 0.913940132 | 0.709328968 | 0.709328968 | 0.755606815 |
| *Triatoma infestans* | SVM | BLOCK | JACCARD | 0.900055578 | 0.716972989 | 0.716972989 | 0.76223043 |
| *Triatoma infestans* | RDF | BLOCK | JACCARD | 0.908981621 | 0.699367355 | 0.699367355 | 0.75481368 |
| *Triatoma infestans* | GAU | BLOCK | JACCARD | 0.910807355 | 0.702094606 | 0.702094606 | 0.749680983 |
| *Triatoma infestans* | SUP | BLOCK | JACCARD | 0.911264654 | 0.70763595 | 0.70763595 | 0.762579867 |
| *Triatoma juazeirensis* | GAU | BOOT | JACCARD | 0.968 | 0.88 | 0.88 | 0.893333333 |
| *Triatoma juazeirensis* | MXD | BOOT | JACCARD | 0.94 | 0.84 | 0.84 | 0.866190476 |
| *Triatoma juazeirensis* | RDF | BOOT | JACCARD | 0.988 | 0.96 | 0.96 | 0.963333333 |
| *Triatoma juazeirensis* | SUP | BOOT | JACCARD | 0.988 | 0.94 | 0.94 | 0.95 |
| *Triatoma juazeirensis* | SVM | BOOT | JACCARD | 0.944 | 0.88 | 0.88 | 0.89 |
| *Triatoma jurbergi* | GAU | BOOT | JACCARD | 1 | 1 | 1 | 1 |
| *Triatoma jurbergi* | MXD | BOOT | JACCARD | 0.6 | 0.6 | 0.6 | 0.8 |
| *Triatoma jurbergi* | RDF | BOOT | JACCARD | 1 | 1 | 1 | 1 |
| *Triatoma jurbergi* | SUP | BOOT | JACCARD | 1 | 1 | 1 | 1 |
| *Triatoma jurbergi* | SVM | BOOT | JACCARD | 1 | 1 | 1 | 1 |
| *Triatoma lenti* | GAU | BOOT | JACCARD | 0.896 | 0.8 | 0.8 | 0.833333333 |
| *Triatoma lenti* | MXD | BOOT | JACCARD | 0.988 | 0.96 | 0.96 | 0.971428571 |
| *Triatoma lenti* | RDF | BOOT | JACCARD | 0.948 | 0.82 | 0.82 | 0.85 |
| *Triatoma lenti* | SUP | BOOT | JACCARD | 0.988 | 0.98 | 0.98 | 0.983333333 |
| *Triatoma lenti* | SVM | BOOT | JACCARD | 0.968 | 0.9 | 0.9 | 0.916666667 |
| *Triatoma maculata* | MXD | BLOCK | JACCARD | 0.784944527 | 0.408320493 | 0.408320493 | 0.621956522 |
| *Triatoma maculata* | SVM | BLOCK | JACCARD | 0.792906489 | 0.426553672 | 0.426553672 | 0.643700935 |
| *Triatoma maculata* | RDF | BLOCK | JACCARD | 0.840246428 | 0.709296353 | 0.709296353 | 0.770956316 |
| *Triatoma maculata* | GAU | BLOCK | JACCARD | 0.826382442 | 0.580722479 | 0.580722479 | 0.70074533 |
| *Triatoma maculata* | SUP | BLOCK | JACCARD | 0.842210726 | 0.661872967 | 0.661872967 | 0.742123984 |
| *Triatoma matogrossensis* | GAU | BOOT | JACCARD | 0.9875 | 0.95 | 0.95 | 0.96 |
| *Triatoma matogrossensis* | MXD | BOOT | JACCARD | 0.85625 | 0.725 | 0.725 | 0.775 |
| *Triatoma matogrossensis* | RDF | BOOT | JACCARD | 1 | 1 | 1 | 1 |
| *Triatoma matogrossensis* | SUP | BOOT | JACCARD | 1 | 1 | 1 | 1 |
| *Triatoma matogrossensis* | SVM | BOOT | JACCARD | 0.98125 | 0.925 | 0.925 | 0.94 |
| *Triatoma melanica* | GAU | BOOT | JACCARD | 0.977777778 | 0.966666667 | 0.966666667 | 0.966666667 |
| *Triatoma melanica* | MXD | BOOT | JACCARD | 0.977777778 | 0.933333333 | 0.933333333 | 0.95 |
| *Triatoma melanica* | RDF | BOOT | JACCARD | 0.888888889 | 0.766666667 | 0.766666667 | 0.8 |
| *Triatoma melanica* | SUP | BOOT | JACCARD | 1 | 1 | 1 | 1 |
| *Triatoma melanica* | SVM | BOOT | JACCARD | 1 | 1 | 1 | 1 |
| *Triatoma melanocephala* | MXD | BLOCK | JACCARD | 0.951575897 | 0.791208791 | 0.791208791 | 0.8175 |
| *Triatoma melanocephala* | SVM | BLOCK | JACCARD | 0.961451247 | 0.822344322 | 0.822344322 | 0.841666667 |
| *Triatoma melanocephala* | RDF | BLOCK | JACCARD | 0.944290142 | 0.765567766 | 0.765567766 | 0.792247744 |
| *Triatoma melanocephala* | GAU | BLOCK | JACCARD | 0.953038415 | 0.8003663 | 0.8003663 | 0.818614719 |
| *Triatoma melanocephala* | SUP | BLOCK | JACCARD | 0.955889654 | 0.822344322 | 0.822344322 | 0.838043478 |
| *Triatoma oliveirai* | GAU | BOOT | JACCARD | 0.95 | 0.9 | 0.9 | 0.933333333 |
| *Triatoma oliveirai* | MXD | BOOT | JACCARD | 0.9 | 0.8 | 0.8 | 0.9 |
| *Triatoma oliveirai* | RDF | BOOT | JACCARD | 0.9 | 0.8 | 0.8 | 0.9 |
| *Triatoma oliveirai* | SUP | BOOT | JACCARD | 0.95 | 0.9 | 0.9 | 0.933333333 |
| *Triatoma oliveirai* | SVM | BOOT | JACCARD | 0.65 | 0.6 | 0.6 | 0.783333333 |
| *Triatoma petrochiae* | GAU | BOOT | JACCARD | 0.985950413 | 0.909090909 | 0.909090909 | 0.917948718 |
| *Triatoma petrochiae* | MXD | BOOT | JACCARD | 0.973553719 | 0.881818182 | 0.881818182 | 0.892732268 |
| *Triatoma petrochiae* | RDF | BOOT | JACCARD | 0.976859504 | 0.881818182 | 0.881818182 | 0.893473193 |
| *Triatoma petrochiae* | SUP | BOOT | JACCARD | 0.98677686 | 0.918181818 | 0.918181818 | 0.924242424 |
| *Triatoma platensis* | SVM | BOOT | JACCARD | 0.949609467 | 0.88 | 0.88 | 0.892427702 |
| *Triatoma platensis* | MXD | BLOCK | JACCARD | 0.9626073 | 0.884997893 | 0.884997893 | 0.894090779 |
| *Triatoma platensis* | SVM | BLOCK | JACCARD | 0.969527059 | 0.917319848 | 0.917319848 | 0.923026316 |
| *Triatoma platensis* | RDF | BLOCK | JACCARD | 0.960157194 | 0.907796039 | 0.907796039 | 0.914771081 |
| *Triatoma platensis* | GAU | BLOCK | JACCARD | 0.964076479 | 0.913232196 | 0.913232196 | 0.919886323 |
| *Triatoma platensis* | SUP | BLOCK | JACCARD | 0.965114236 | 0.922081753 | 0.922081753 | 0.92710177 |
| *Triatoma pseudomaculata* | MXD | BLOCK | JACCARD | 0.939559575 | 0.766788127 | 0.766788127 | 0.800016776 |
| *Triatoma pseudomaculata* | SVM | BLOCK | JACCARD | 0.940160015 | 0.773643204 | 0.773643204 | 0.808935411 |
| *Triatoma pseudomaculata* | RDF | BLOCK | JACCARD | 0.927830976 | 0.742682782 | 0.742682782 | 0.780347158 |
| *Triatoma pseudomaculata* | GAU | BLOCK | JACCARD | 0.938005613 | 0.76501659 | 0.76501659 | 0.799035135 |
| *Triatoma pseudomaculata* | SUP | BLOCK | JACCARD | 0.942801125 | 0.767617609 | 0.767617609 | 0.804874758 |
| *Triatoma rubrofasciata* | GAU | BOOT | JACCARD | 0.910204082 | 0.742857143 | 0.742857143 | 0.784642857 |
| *Triatoma rubrofasciata* | MXD | BOOT | JACCARD | 0.840816327 | 0.614285714 | 0.614285714 | 0.706493506 |
| *Triatoma rubrofasciata* | RDF | BOOT | JACCARD | 0.881632653 | 0.671428571 | 0.671428571 | 0.750248918 |
| *Triatoma rubrofasciata* | SUP | BOOT | JACCARD | 0.920408163 | 0.785714286 | 0.785714286 | 0.810515873 |
| *Triatoma rubrofasciata* | SVM | BOOT | JACCARD | 0.918367347 | 0.771428571 | 0.771428571 | 0.80234127 |
| *Triatoma rubrovaria* | MXD | BLOCK | JACCARD | 0.961277013 | 0.886240703 | 0.886240703 | 0.892512077 |
| *Triatoma rubrovaria* | SVM | BLOCK | JACCARD | 0.956259735 | 0.89198783 | 0.89198783 | 0.897588523 |
| *Triatoma rubrovaria* | RDF | BLOCK | JACCARD | 0.960926318 | 0.89198783 | 0.89198783 | 0.895962733 |
| *Triatoma rubrovaria* | GAU | BLOCK | JACCARD | 0.96164668 | 0.897734956 | 0.897734956 | 0.903083028 |
| *Triatoma rubrovaria* | SUP | BLOCK | JACCARD | 0.961226726 | 0.897734956 | 0.897734956 | 0.903083028 |
| *Triatoma sherlocki* | GAU | BOOT | JACCARD | 0.822222222 | 0.766666667 | 0.766666667 | 0.783333333 |
| *Triatoma sherlocki* | MXD | BOOT | JACCARD | 0.944444444 | 0.833333333 | 0.833333333 | 0.875 |
| *Triatoma sherlocki* | RDF | BOOT | JACCARD | 0.916666667 | 0.766666667 | 0.766666667 | 0.825 |
| *Triatoma sherlocki* | SUP | BOOT | JACCARD | 0.944444444 | 0.833333333 | 0.833333333 | 0.875 |
| *Triatoma sherlocki* | SVM | BOOT | JACCARD | 0.777777778 | 0.633333333 | 0.633333333 | 0.716666667 |
| *Triatoma sordida* | MXD | BLOCK | JACCARD | 0.888560341 | 0.679581914 | 0.679581914 | 0.748213946 |
| *Triatoma sordida* | SVM | BLOCK | JACCARD | 0.89593649 | 0.691523788 | 0.691523788 | 0.744197465 |
| *Triatoma sordida* | RDF | BLOCK | JACCARD | 0.900996448 | 0.717715088 | 0.717715088 | 0.768732398 |
| *Triatoma sordida* | GAU | BLOCK | JACCARD | 0.897475877 | 0.693483364 | 0.693483364 | 0.742735161 |
| *Triatoma sordida* | SUP | BLOCK | JACCARD | 0.900996448 | 0.717715088 | 0.717715088 | 0.768732398 |
| *Triatoma tibiamaculata* | MXD | BLOCK | JACCARD | 0.903588441 | 0.734848485 | 0.734848485 | 0.787994891 |
| *Triatoma tibiamaculata* | SVM | BLOCK | JACCARD | 0.96115272 | 0.871212121 | 0.871212121 | 0.885093168 |
| *Triatoma tibiamaculata* | RDF | BLOCK | JACCARD | 0.907175448 | 0.740530303 | 0.740530303 | 0.770833333 |
| *Triatoma tibiamaculata* | GAU | BLOCK | JACCARD | 0.940857438 | 0.806818182 | 0.806818182 | 0.830357143 |
| *Triatoma tibiamaculata* | SUP | BLOCK | JACCARD | 0.960571625 | 0.827651515 | 0.827651515 | 0.844907407 |
| *Triatoma vandae* | GAU | BOOT | JACCARD | 1 | 1 | 1 | 1 |
| *Triatoma vandae* | MXD | BOOT | JACCARD | 1 | 1 | 1 | 1 |
| *Triatoma vandae* | RDF | BOOT | JACCARD | 1 | 1 | 1 | 1 |
| *Triatoma vandae* | SUP | BOOT | JACCARD | 1 | 1 | 1 | 1 |
| *Triatoma vandae* | SVM | BOOT | JACCARD | 1 | 1 | 1 | 1 |
| *Triatoma vitticeps* | MXD | BLOCK | JACCARD | 0.879133565 | 0.686800172 | 0.686800172 | 0.757704707 |
| *Triatoma vitticeps* | SVM | BLOCK | JACCARD | 0.861895235 | 0.591408269 | 0.591408269 | 0.707634902 |
| *Triatoma vitticeps* | RDF | BLOCK | JACCARD | 0.851256989 | 0.584732989 | 0.584732989 | 0.678663737 |
| *Triatoma vitticeps* | GAU | BLOCK | JACCARD | 0.834264095 | 0.496231697 | 0.496231697 | 0.664781746 |
| *Triatoma vitticeps* | SUP | BLOCK | JACCARD | 0.878901819 | 0.66580534 | 0.66580534 | 0.737739872 |
| *Triatoma williami* | GAU | BOOT | JACCARD | 0.936734694 | 0.814285714 | 0.814285714 | 0.840277778 |
| *Triatoma williami* | MXD | BOOT | JACCARD | 0.946938776 | 0.828571429 | 0.828571429 | 0.842857143 |
| *Triatoma williami* | RDF | BOOT | JACCARD | 0.914285714 | 0.8 | 0.8 | 0.833333333 |
| *Triatoma williami* | SUP | BOOT | JACCARD | 0.936734694 | 0.857142857 | 0.857142857 | 0.869642857 |
| *Triatoma williami* | SVM | BOOT | JACCARD | 0.928571429 | 0.828571429 | 0.828571429 | 0.844642857 |
| *Triatoma wygodzinskyi* | GAU | BOOT | JACCARD | 0.966666667 | 0.9 | 0.9 | 0.925 |
| *Triatoma wygodzinskyi* | MXD | BOOT | JACCARD | 0.866666667 | 0.766666667 | 0.766666667 | 0.783333333 |
| *Triatoma wygodzinskyi* | RDF | BOOT | JACCARD | 1 | 1 | 1 | 1 |
| *Triatoma wygodzinskyi* | SUP | BOOT | JACCARD | 1 | 1 | 1 | 1 |
| *Triatoma wygodzinskyi* | SVM | BOOT | JACCARD | 0.988888889 | 0.966666667 | 0.966666667 | 0.975 |


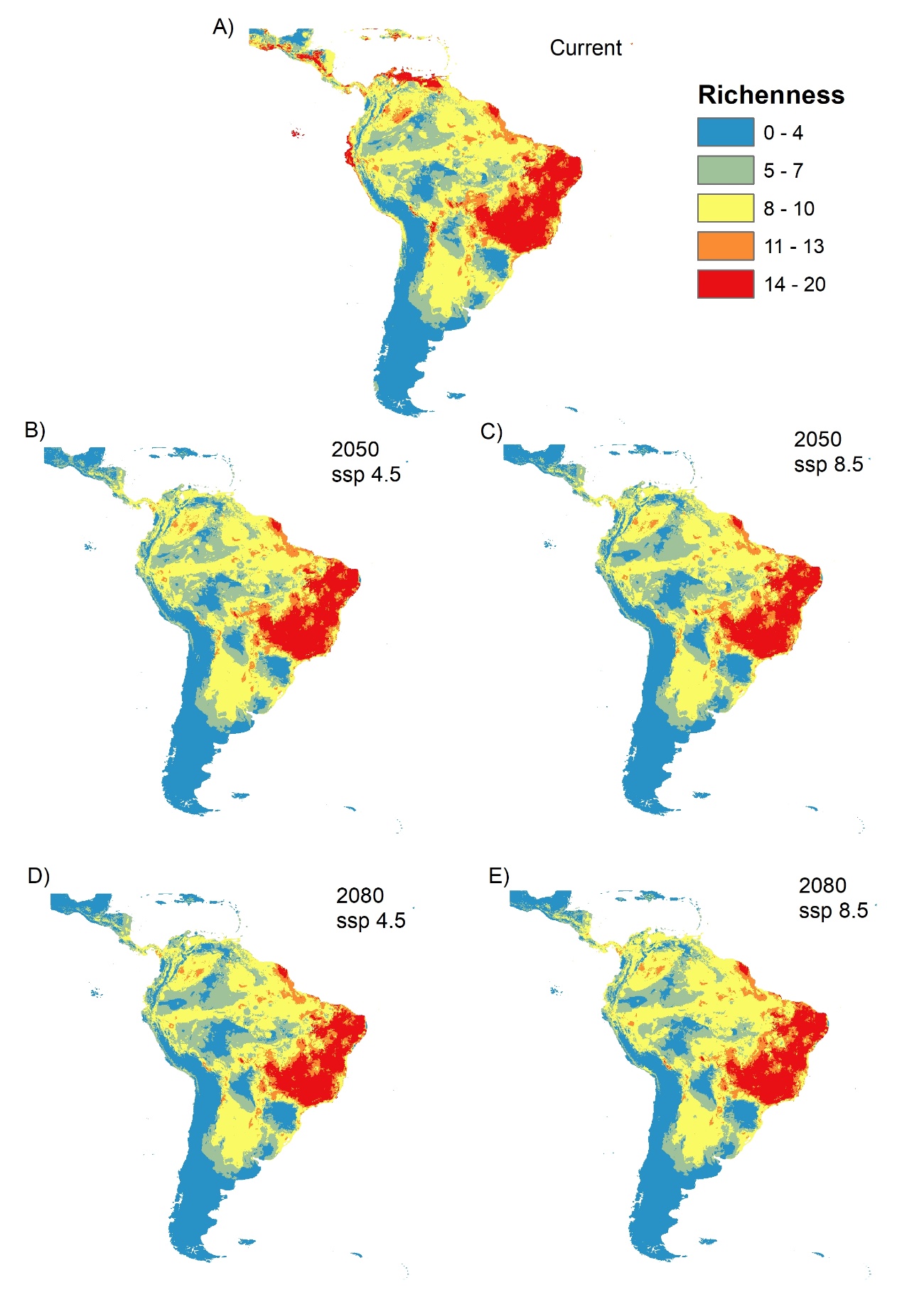


**Figure S1**. Richness distribution model for triatomine species richness of triatomines in the present (A), moderate-warming scenario (SSP2-4.5) future in 2050 with mild changes (B), high-emission scenariofuture in 2050 (SSP5-8.5) with significant changes (C), moderate-warming scenario (SSP2-4.5)future in 2080 with mild changes (D), high-emission scenarioand future in 2080 with significant changes (E).
